# Are Corporate Political Actors Aware of the Health Risks Associated With Their Products? Comment on "Corporate Political Activity: Taxonomies and Model of Corporate Influence on Public Policy"

**DOI:** 10.34172/ijhpm.9104

**Published:** 2025-08-17

**Authors:** Emanuel Orozco

**Affiliations:** Health Systems Research Centre, National Institute of Public Health, Morelos, Mexico.

**Keywords:** Corporate Political Activity, Health Policy, Non-communicable Diseases

## Abstract

The analytical model proposal by Ulucanlar et al for the analysis of corporate political activity (CPA) establishes that corporate actors are aware of the high incidence of non-communicable diseases (NCDs) associated with their products and protect themselves against the consequences. This model helps to identify the strategies used by various corporate entities to influence public policies and protect their interests. The CPA analytical model applies a critical approach to commercial determinants of health that allows us to understand how corporate actors take advantage of their systems-based management of power. Due to its inherent complexity, this analysis presents several unanswered questions requiring along with theoretical and empirical challenges. The situation described in this commentary points to the importance of monitoring the CPA at a global and local level, identifying opportunities that favor the regulation of political influence, to reduce the influence of the commercial determinants of health on the NCDs.

 From a public health perspective, the influence that the industrial mode of production and global commerce has had on morbidity and mortality of non-communicable diseases (NCDs) has become a global challenge. When searching for explanatory mechanisms of this influence the analysis of the corporate political activity (CPA) represents a promising approach, given its theoretical relevance that arises from the discussion on the commercial determinants of health.^1^ One of the main causal agents of this situation is the manufacture and marketing of harmful products for health by the tobacco, alcohol and gambling industries, as well as those involved in the production of ultra-processed drinks and foods. These industries have been conceptualized as unhealthy commodity industries (UCIs). The analysis of CPA delves into the mechanisms that these industries utilize to block or delay the implementation of public policies. This analysis resorts to analytical models and consistent research techniques to support researchers, decision-makers, activists, and legislators in identifying potential conflicts of interest of corporative stakeholders in the political arena in order to implement and protect healthy policies.

 The article by Ulucanlar et al^1^ on CPA represents a great effort to put into perspective the research work carried out by a broad team of people with an important trajectory on this topic at a global level. One of the article’s most relevant contributions is the formulation of an evidence-based model to document CPA where it occurs. The article has a strong conceptual component, and at the same time it defines a set of framing and action taxonomies used by corporations to act politically. It also analyzes the strengths and weaknesses of the CPA in terms of the model developed. Apart from their conceptual work, the text presents an innovative methodology that rigorously combines different information sources and analytical procedures. Through this, authors pretend to draw the attention of research communities, civil organizations and government decision-makers with respect to the influence of tobacco, alcohol and gambling industries, as well as those involved in the production of ultra-processed drinks and foods, on the global profiles of morbidity and mortality due to NCDs.

## Global Health Issues

 The analysis of the CPA results effective for establishing that corporate actors are aware of the high incidence of NCDs associated with their products and deliberately mobilize political and economic resources to protect themselves against the consequences. Thus, in Ulucanlar and colleagues’ opinion, the CPA’s impact is related to a substantial proportion of the 41 million annual deaths due to NCDs. These diseases are largely attributable to the consumption of the UCIs’ products and to the strategies the corporative sector displays to defend their interests. The analysis of CPA has been strengthened during the last decade, showing, through numerous case studies, that global public health faces the threat of becoming contingent upon pecuniary interests.^2^ This stems from the political influence that the UCIs exert on governments almost worldwide.^3^ It has been precisely the expansion of those interests what has promoted the development of corporate strategies to influence governmental decision-making.

 The application of the analytical model of the CPA provides timely guidance on the context and the processes directed to the political influence of the UCIs and their specific strategies, thus showing its potential outreach at a practical level. In two empirical studies we documented different implications of the CPA analytical model. The first case showed that in Mexico, the industry of sugar sweetened beverages tried to block taxation mainly by highlighting the important role to the economy that this industry has, arguing the negative effects in sales and unemployment, and mobilizing the industry’s relationships with governmental bodies and civil society actors to promote corporate social responsibility and gain public support.^4^ The second example documented that food industry actors in Colombia, mainly at the national level, corporative actors operated as incentive providers to Colombia’s government officials in the executive and legislative branches. This analysis shows that the incentives of the food industry were useful to influence the processes of formulation and implementation of food and nutritional public policies.^5^

 The analytical model of APC underscores situational elements that show the display of framing and action political strategies purposely directed to promote the marketing and consumption of products manufactured by the UCIs. Some of the more significant strategies include the following: creation of misleading contents about their products, state substitution, questioning scientific evidence, access to decision-makers, and engaging in lawsuits.^6^ One-pointedly or conjointly, these strategies are intended to create a narrative framework that fosters a good impression of the corporations among different audiences, profiting from the available means, as well as labeling those who question them as exaggerated or manipulative. Their goals are to block public policies or to delay their implementation, thus creating conditions that benefit their interests, influencing the public decision-making processes in different ways.^6^

## Extents of the Corporate Political Activity

 The CPA analytical model implies a critical approach to commercial determinants of health because it allows us to understand how corporate actors take advantage of their systems-based understanding of power. Given the multi-layered nature of policy-making, several structural interventions are needed to counteract the main extents of the CPA that can be observed at the social, political and economic levels:

 Social. One of the CPA’S most outstanding effects is its capacity to modify the perception of lifestyles, promoting risk behaviors and discouraging healthy habits. In this respect, it is noteworthy that the consequences of the consumption of the UCIs’ products in terms of health are, in their narrative, attributed to the people’s inability to self-regulate. Therefore, they establish that modulation reduces the risks, and that it is through educational processes, not regulatory policies, how individual decisions to avoid health damages can be steered to avoid health damages.^7^ Another notable feature of these industries’ discourse is the alleged limitation of freedoms that consumption regulation might constitute, highlighting that the State must not intervene in presumably individual and private decisions.

 Political. Contents released through different channels rendering a positive representation of the UCIs, accompanied by an allegedly political neutrality, are promoted by a variety of actors from the social, commercial and governmental sectors. This diversity of participants focuses their efforts on blocking or delaying public policies aimed at regulating the consumption of products marketed by the UCIs. The actors involved participate in different spheres such as academic, political, governmental and civil, and they include a wide range of representatives. Some of the areas where the CPA’s interference has been more widely documented include fiscal policies concerning unhealthy products, warning labeling, and non-polluting packaging of products.^8^

 Economic. Backing up the commercial expansion of the corporations, there is the argument that they invest capital, generate jobs, and aim to innovate their processes to increase their economic capacity. This argument operates within the UCIs as a facade that conceals health-related risks and harms derived from the marketing and consumption of their products.^9^ One of the major risks stemming from the economic influence of these industries is the system of exchanging favors between commercial agents and political groups, as well as the UCIs’ financing of sports teams, NGOs, schools and, when legal vacuums allow it, even political parties. They also get involved in the placement of commercial agents in government agencies to expand their influence, phenomenon known as “revolving doors.”^10^

 Nevertheless the comprehensiveness of the CPA analytical model of Ulucanlar et al, could present a series of reasonable questions to solve. Firstly, its inherent complexity could present theoretical and empirical challenges and, secondly, imply a major consideration of the “political marketplace” in terms of dynamics and outcomes.^11^ To advance in this direction it has been proposed a deeper analysis of attitudes and perceptions of CPA under a societal perspective. While there is so much analytical emphasis on lobbying and political campaigns, it should be considered that not all corporations take political corporative advantage, and some commercial actors could deny participating politically as the CPA analytical model suggests. Finally, it is necessary to consider how society understands the problem, and to generate more evidence on CPA strategies’ variability and cultural adaptability.^11^

 The questions described point to the importance of delving into and monitoring the CPA at a global and local level. This will help to identify opportunities that enhance the regulation of the political incidence activities that support their interests and thus, to reduce the influence of the commercial determinants of health with respect to NCDs. Socially, there is a long way to go in order to modify perceptions on the consumption of products that can harm health and to document other health risks related to other corporate industries that could operate in a similar way. Politically, it is necessary to identify the UCIs’ practices that weaken the governments’ governance and regulatory capacity so as to prevent the predominance of those industries commercial and political agendas, as well as the disinformation on health risks and harms related to the consumption of their products. When political and economic interests are combined, there arise power asymmetries that weaken social and political mobilization in pursuit of healthier societies.

## Ethical issues

 Not applicable.

## Conflicts of interest

 Author declares that he has no conflicts of interest.
